# Panel‐based whole exome sequencing identifies novel mutations in microphthalmia and anophthalmia patients showing complex Mendelian inheritance patterns

**DOI:** 10.1002/mgg3.329

**Published:** 2017-08-21

**Authors:** Marina Riera, Ana Wert, Isabel Nieto, Esther Pomares

**Affiliations:** ^1^ Departament de Genètica Institut de Microcirurgia Ocular (IMO) Barcelona Spain; ^2^ Departament d'Oftalmologia Pediàtrica, Estrabisme i Neuroftalmologia Institut de Microcirurgia Ocular (IMO) Barcelona Spain; ^3^ Departament de Còrnia, Cataracta i Cirurgia Refractiva Institut de Microcirurgia Ocular (IMO) Barcelona Spain

**Keywords:** Anophthalmia, microphthalmia, ophthalmology, whole exome sequencing

## Abstract

**Background:**

Microphthalmia and anophthalmia (MA) are congenital eye abnormalities that show an extremely high clinical and genetic complexity. In this study, we evaluated the implementation of whole exome sequencing (WES) for the genetic analysis of MA patients. This approach was used to investigate three unrelated families in which previous single‐gene analyses failed to identify the molecular cause.

**Methods:**

A total of 47 genes previously associated with nonsyndromic MA were included in our panel. WES was performed in one affected patient from each family using the AmpliSeq^TM^ Exome technology and the Ion Proton^TM^ platform.

**Results:**

A novel heterozygous *OTX2* missense mutation was identified in a patient showing bilateral anophthalmia who inherited the variant from a parent who was a carrier, but showed no sign of the condition. We also describe a new *PAX6* missense variant in an autosomal‐dominant pedigree affected by mild bilateral microphthalmia showing high intrafamiliar variability, with germline mosaicism determined to be the most plausible molecular cause of the disease. Finally, a heterozygous missense mutation in *RBP4* was found to be responsible in an isolated case of bilateral complex microphthalmia.

**Conclusion:**

This study highlights that panel‐based WES is a reliable and effective strategy for the genetic diagnosis of MA. Furthermore, using this technique, the mutational spectrum of these diseases was broadened, with novel variants identified in each of the *OTX2*,*PAX6*, and *RBP4* genes. Moreover, we report new cases of reduced penetrance, mosaicism, and variable phenotypic expressivity associated with MA, further demonstrating the heterogeneity of such disorders.

## Introduction

Microphthalmia and anophthalmia (MA) are the most severe congenital eye malformations, affecting approximately 1 in 7,000 and 1 in 30,000 births, respectively (Morrison et al. 2002; Shah et al. 2011; Williamson and Fitzpatrick 2014). Microphthalmia refers to a reduction in the size of the ocular globe (axial length <21 mm in adults and <19 mm in a 1‐year‐old child), whereas anophthalmia is defined as the complete absence of the eye. These defects can occur uni‐ or bilaterally, and can be part of a syndromic disease (Bardakjian et al. [Link]). Microphthalmia can be isolated (simple microphthalmia) or associated with other eye anomalies such as coloboma and congenital cataracts (complex microphthalmia).

Epidemiological studies have shown that both heritable and environmental factors can be responsible for MA, with genetics being the most common cause (Bermejo and Martinez‐Frias 1998; Verma and Fitzpatrick 2007; Chassaing et al. 2014). In fact, over recent years, several chromosomal abnormalities and point mutations have been identified as being related to MA. In particular, chromosomal rearrangements have been found to mainly be associated with syndromic MA, whereas single‐nucleotide variants have been identified in both syndromic and nonsyndromic forms (Bardakjian and Schneider 2011; Slavotinek 2011). All the Mendelian inheritance patterns have been reported for these pathologies; however, their prediction is complicated as most cases are sporadic, and the occurrence of de novo mutations, mosaicism, and incomplete penetrance makes genetic counseling an even greater challenge (Morrison et al. 2002). Of the monogenic causes of MA, heterozygous mutations in *SOX2* (OMIM *184429) are the most prevalent, accounting for 10–40% of bilateral MA cases (Williamson and Fitzpatrick [Link]; Ragge et al. 2005b; Reis et al. 2010). In addition to *SOX2*, more than 20 other genes have been identified as being responsible for MA, although most explain only a small fraction of cases (<1%) (Williamson and Fitzpatrick 2014; Plaisancie et al. 2016b).

*OTX2* (OMIM * 600037), a homeobox‐containing transcription factor known to play a key role in vertebrate brain development, has been shown to be associated with a wide range of ocular disorders, including MA (Acampora and Simeone 1999; Chassaing et al. 2014). This gene accounts for approximately 3% of bilateral MA, with de novo heterozygous loss‐of‐function mutations being the most common cause (Wyatt et al. 2008). However, several cases of incomplete penetrance have been previously described, and it is estimated that up to 35% of *OTX2* patients inherit the mutation from a carrier parent without the condition (Ragge et al. 2005a; Schilter et al. 2011; Gerth‐Kahlert et al. 2013).

*PAX6* (OMIM * 607108) encodes a transcription factor that plays a central role in ocular and brain development, and was identified as the gene associated with aniridia (absence or hypoplasia of the iris), with more than 300 mutations currently described (Glaser et al. 1992). MA is also part of the *PAX6*‐associated phenotypic spectrum, but has only been reported in a very small number of families (Deml et al. 2016).

*RBP4* (OMIM * 180250) encodes a serum retinol‐binding protein that is responsible for transportation of retinol from the liver stores to peripheral tissues, including the placenta and the fetal eye (Blaner 1989; Zanotti and Berni 2004; D'Ambrosio et al. 2011). A previous study identified different missense mutations in *RBP4* gene in three unrelated families affected by MA, the phenotypes of which exhibited dominant inheritance, incomplete penetrance, and skewed maternal transmission (Chou et al. 2015).

Available molecular genetic testing of MA patients, including single‐gene sequencing, copy number variation analysis, and chromosome microarray studies, is able to identify the molecular cause of the condition in almost 80% of individuals with bilateral anophthalmia or severe microphthalmia. However, in typical MA cohorts, with a mix of laterality and severity, this percentage decreases to approximately 20% (Gerth‐Kahlert et al. 2013; Chassaing et al. 2014). Recently, next‐generation sequencing (NGS) technologies, including multi‐gene panels or more broad‐based NGS strategies such as whole exome sequencing (WES) or whole genome sequencing (WGS), have revolutionized genetic diagnosis of several heterogeneous inherited diseases, including MA disorders (Jimenez et al. 2011; Deml et al. 2014, 2016; Slavotinek et al. 2015). In this study, we used WES to examine 47 genes in three unrelated MA families. We identified novel missense variants in the *OTX2*,* PAX6*, and *RBP4* candidates, describing complex inheritance patterns based on incomplete penetrance and mosaicism, and showing, in some cases, extreme intrafamiliar phenotypic variability.

## Materials and Methods

### Ethical compliance

This study has been approved by the ethics committee of Institut de Microcirurgia Ocular (Barcelona, Spain).

### Patients

Three unrelated families affected by MA were included in this study. One family (MA_1) originated from the Arabian Peninsula, whereas the other two (MA_2 and MA_3) were from Spain. Clinical diagnoses were established at the Institut de Microcirurgia Ocular and were based on standard ophthalmic evaluations. In the cases of microphthalmia, best corrected visual acuity and axial length (measured using biometrics in adults and ultrasound scanning in children) were determined, and cycloplegic refraction and retinography were carried out. For the *PAX6*‐affected patients, optical coherence tomography (OCT) was additionally performed. In the cases of anophthalmia, magnetic resonance imaging (MRI) and ultrasound examinations were used to assess the phenotype.

Peripheral blood from patients and their relatives was collected in EDTA‐containing tubes, and automated extraction of genomic DNA was performed using the Mag‐Bind Blood DNA HDQ purification system (Omega Bio‐Tek, Norcross, GA, USA). All procedures used in this study were performed in accordance with the Declaration of Helsinki and all participants were informed of the purpose and procedures of the study (written consent was obtained from each individual).

### Gene panel design, whole exome sequencing, and data processing

A total of 47 genes previously associated with nonsyndromic microphthalmia and anophthalmia were included in our MA panel (Table S1). Genes were selected according to the information available in OMIM (http://www.omim.org), Orphanet (http://www.orphanet.net), and Pubmed databases (http://www.ncbi.nlm.nih.gov/pubmed/).

WES was performed in one affected patient from each family using libraries designed and constructed by the AmpliSeq^TM^ Exome technology (Thermo Fisher Scientific, Waltham, MA, USA). Generated amplicons were genotyped with the Ion Proton^TM^ platform (Thermo Fisher Scientific), following the manufacturer's instructions. Sequences were aligned against the reference genome (GRCh37/hg19) using TMAP Alignment (Thermo Fisher Scientific). WES was performed in collaboration with a private company (NIMGenetics, Madrid, Spain), which provided the BAM, BAI, and FASTQ files, as well as the VCF and TSV files that contained a compilation of all variants detected using the Ion Reporter software (Thermo Fisher Scientific). Moreover, the company supplied a TSV file that specifically included the variants identified in the 47 candidates (after running a custom pipeline), and a document that detailed the coverage data of the panel, specifying the number of reads of each amplicon. The coverage information obtained from the three samples was used to measure the average depth and the percentage of coverage >20× of each gene.

### Identification and analysis of pathogenic variants

Variants that were detected in genes included in the MA panel were filtered according to coverage (≥15×), minor allele frequency (≤0.01), and deleterious potential. All resulting variants were contrasted with the polymorphism and mutation databases gnomAD (http://gnomad.broadinstitute.org/), ExAC (http://exac.broadinstitute.org/), ESP (http://evs.gs.washington.edu/EVS/), HGMD (http://www.hgmd.cf.ac.uk), and Uniprot (http://www.uniprot.org/). The pathogenicity of missense changes was evaluated using the following in silico predictors: SIFT, PolyPhen‐2, LRT, MutationTaster, MutationAssessor, FATHMM, MetaSVM, and CONDEL. Moreover, nucleotide conservation was evaluated using the PhastCons and PhyloP programs. Sanger sequencing was performed to confirm the putative pathogenic variants obtained after WES genotyping, and mutation segregation analysis was carried out when relative's samples were available. Finally, for each potentially pathogenic variant, 180 healthy control individuals were analyzed.

## Results

### Genetic analysis

**Figure 1:**
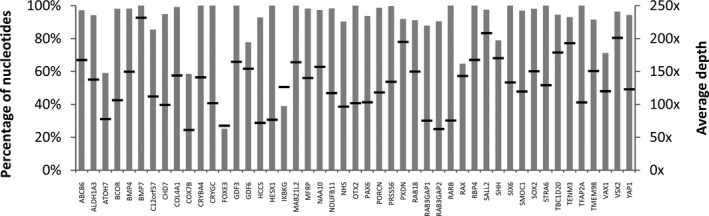
Coverage statistics of coding regions of genes included in the MA panel. The percentage of nucleotides with >20× depth coverage per gene is shown (gray bars). Black lines represent the average depth in each case.

The Ion Ampliseq^TM^ Exome technology and Ion Proton^TM^ platform allowed for the capturing, amplification, and sequencing of more than 97% of the coding regions of >19,000 genes. The generated data were filtered to obtain coverage information and variants of the 47 genes included in the MA panel (the full list of genes is given in Table S1). In particular, 812 amplicons were found to cover 96.6% of the coding regions and flanking exon/intron boundaries (around 50 bp) of the MA genes. After the analysis, only 5.4% of the amplicons had coverage of <20×. On average, depth coverage of the genes was 131×, with values that ranged from 61× (*COX7B*) to 231× (*BMP7*). Most of the candidates (78.8%, 37/47) displayed an average depth of >100×. A schematic representation of the percentage of nucleotides covered at >20×, as well as the mean value for each gene, is shown in Figure 1.

### New missense mutation in *OTX2* showing incomplete penetrance

**Figure 2:**
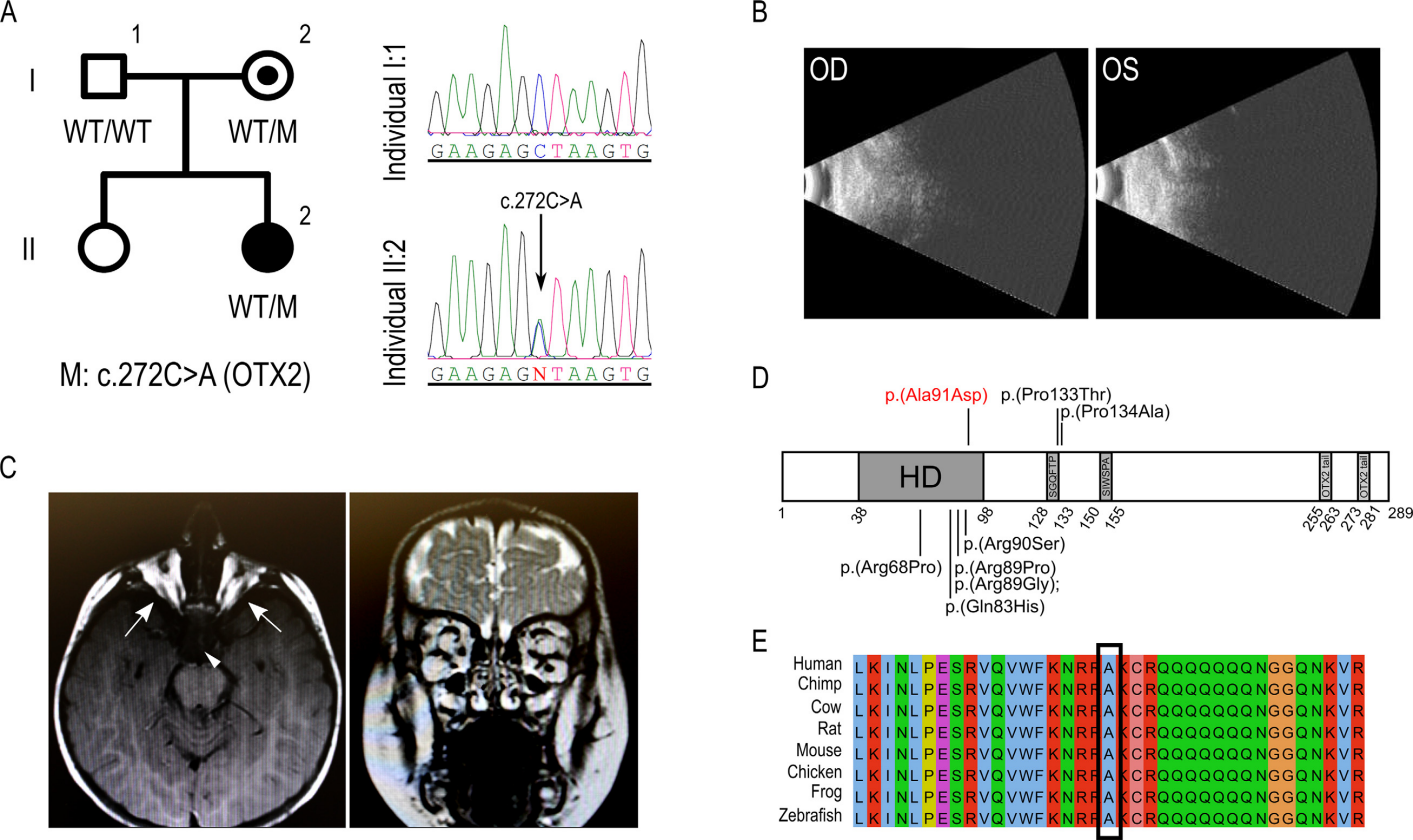
New *OTX2* missense variant associated with anophthalmia. (A) Pedigree of family MA_1 showing the cosegregation analysis of the *OTX2* variant. DNA chromatograms for patient II:2 and her father (I:1) showing the position of the variant. Note that the unaffected mother also carried the mutation. (B) Ultrasound scan of both eyes showing complete absence of ocular globes. (C) Axial and coronal MRI images revealing absence of the optic nerves and chiasm (the triangle depicts the region where optic chiasm should be), but presence of extraocular muscles in individual II:2 (arrows). (D) Schematic representation of the OTX2 protein, showing the homeodomain (HD) as well as the SGQFTP, SIWSPA, and OTX2 tail motifs. The position of the new p.(Ala91Asp) mutation is depicted in red, whereas previously described MA missense variants are shown in black (according to HGMD database). (E) Amino acid alignment of OTX2 proteins from different species. The affected residue is conserved in all species.

Pedigree MA_1 was a family from Arabian Peninsula with a 1‐year‐old child showing bilateral anophthalmia (II:2), and no previous family history of MA (Fig. 2A). Ultrasound scans showed complete absence of the ocular globes; while MRI revealed that the optic nerve and chiasm were absent and that the extraocular muscles were present (Table 1 and Fig. 2B,C). The same exploration showed no other cranioencephalic alterations and the patient did not exhibit other systemic anomalies.

**Table 1:** Summary of genetic and clinical features of microphthalmia/anophthalmia patients included in this study

Family	Gene	Genomic change (GRCh37/hg19)	Nucleotide change	Protein change	Status	Pattern of inheritance	Patient	Gender	Age	Ocular phenotype	BCVA	Axial length (mm)	Extraocular phenotype
MA_1	*OTX2*	g.57269051G>T	c.272C>A	p.(Ala91Asp)	Het	Autosomal dominant with incomplete penetrance	III:2	Female	1	Bilateral anophthalmia, absence of optic nerves and quiasm	NLP	NA	None
MA_2	*PAX6*	g.31824262C>G	c.131G>A	p.(Arg44Pro)	Het	Autosomal dominant showing gonosomal mosaicism	III:3	Female	43	Mild bilateral microphthalmia, bilateral congenital cataract, nystagmus, foveal hypoplasia	20/65; 20/65	20.63/20.14	None
							III:5	Female	38	Mild bilateral microphthalmia, bilateral congenital cataract, nystagmus, foveal hypoplasia	20/65; 20/100	20.85/20.75	None
							IV:3	Female	4	Shortened axial length, unilateral central cataract, nystagmus, strabismus, hyperopia (+5)	20/100; 20/100	18.89/19.00	None
							IV:4	Male	1	Unilateral central and cortical cataract, hyperopia (+4)	20/380; 20/380^a^	19.00/19.00	None
MA_3	*RBP4*	g.95353754A>T	c.394T>A	p.(Tyr132Asn)	Het	Autosomal dominant showing skewed maternal transmission, de novo phenomenon or incomplete penetrance	III:3	Male	64	Bilateral microphthalmia and coloboma	20/2000; 20/2000	‐	None

BCVA, best corrected visual acuity; Het, heterozygous; NLP, no light perception; NA, not applicable.

a Tested with Teller Acuity Cards.

In a previous study, we screened *SOX2* for mutations in the affected patient, this gene being the most frequent molecular cause of severe bilateral eye malformations, such as anophthalmia. After screening for point mutations and gross rearrangements in *SOX2*, the gene was discarded as the genetic cause. Here, analysis of the MA panel in the patient revealed a heterozygous missense mutation in *OTX2*, c.272C>A (p.(Ala91Asp)) (NG_008204.1, NM_001270524.1, NP_001257453.1) (Fig. 2A,D). This novel variant, which has not been previously described in any public or private database, was predicted as being deleterious by several missense prediction algorithms (Table 2), and was not found in any of the 180 control individuals (21 of them from the Arabian Peninsula). The segregation study from both unaffected parents identified the mother (I:2) as a likely incomplete penetrant carrier as she also presented the pathogenic variant (Fig. 2A). At the protein level, the mutation affected a highly conserved alanine residue of the OTX2 homeodomain DNA‐binding motif, in which other heterozygous missense variants have been previously associated to ocular malformations (Fig. 2D,E).

**Table 2:** Pathogenicity predictions for new missense mutations

Family ID	Gene	Nucleotide change	Protein change	PhastCons	PhyloP	SIFT	Poly Phen2	LRT	Mutation taster	Mutation assessor	FATHMM	MetaSVM	CONDEL	Reference
MA_1	*OTX2*	c.272C>A	p.(Ala91Asp)	1	5.61	D	D	D	D	H	D	D	D	This study
MA_2	*PAX6*	c.131G>C	p.(Arg44Pro)	1	5.61	D	D	N	D	H	D	D	D	This study
MA_3	*RBP4*	c.394T>A	p.(Tyr132Asn)	1	5.13	D	D	D	D	M	D	D	D	This study

D, deleterious or disease causing; H, high; M, medium; N, neutral.

### Occurrence of germline mosaicism in a family carrying a novel *PAX6* mutation

**Figure 3:**
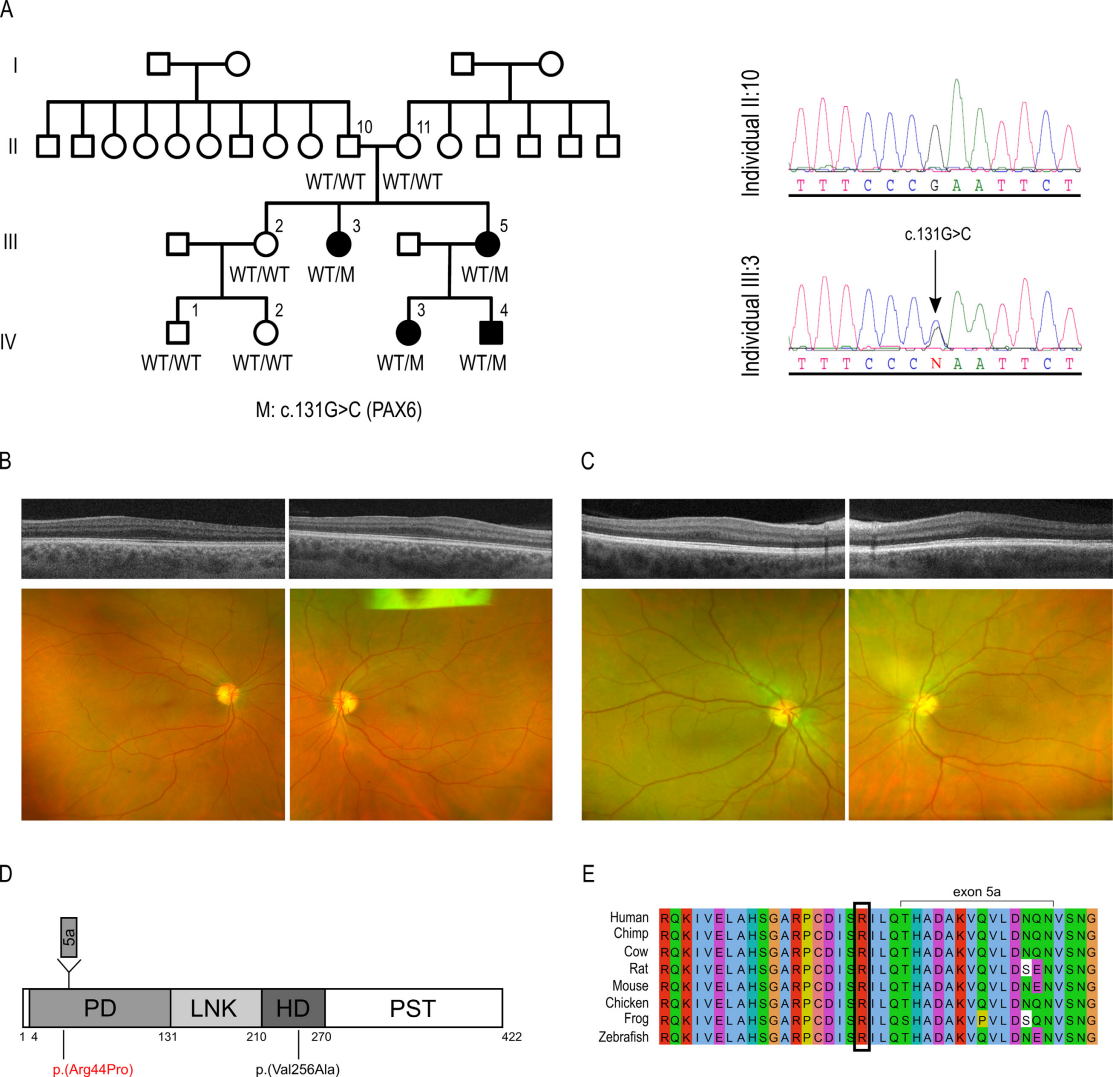
*PAX6* mutation causing microphthalmia, congenital cataracts, and foveal hypoplasia. (A) MA_2 family pedigree and *PAX6* variant cosegregation and chromatogram of the nucleotide heterozygous variant identified in *PAX6*. (B, C) OCT and fundus examination of patients III:3 and III:5, respectively. Note that OCT shows bilateral foveal hypoplasia in both cases. (D) Schematic representation of the PAX6 protein, showing the paired domain (PD), linker region (LNK), homeodomain (HD), and proline‐serine‐threonine‐rich transactivation domain (PST). Protein region codified by exon 5a is also shown. The position of the new p.(Arg44Pro) mutation is depicted in red, whereas the previously described p.(Val256Ala) missense MA variant is shown in black. (E) Alignment of PAX6 of eight different vertebrate species, showing a high conservation of the affected arginine.

Spanish family MA_2 contained two affected members (III:3 and III:5) with mild bilateral microphthalmia, congenital cataracts, and vertical nystagmus (Table 1 and Fig. 3A). The two patients underwent cataract surgery and intraocular lens implantation at the age of 18 (III:5) and 23 (III:3) years. Interestingly, fundus examinations of both individuals appeared normal; however, OCT revealed foveal hypoplasia (Fig. 3B,C).

When this family was firstly studied at our center in 2013, only patients III:3 and III:5 showed clinical features compatible with microphthalmia, with no other family member suspected of having an eye disorder. At that time, patient III:5 had a 1‐year‐old child (IV:3) who occasionally showed very mild nystagmus, but in whom no other conclusive evidence of microphthalmia was found. Within this context, an autosomal recessive pattern was thought to be the most probable mode of inheritance in the family. Thus, subsequent genetic analysis was focused on direct sequencing of all coding regions of two recessive MA candidates, *MFRP* and *PRSS56*. However, no pathogenic variant was identified in these genes. More recently, our genetic analysis using the MA panel showed that patient III:5 carried a heterozygous missense mutation, c.131G>C (p.(Arg44Pro)), in the *PAX6* gene (NG_008679.1, NM_000280.3, NP_000271.1) (Fig. 3A,D). This variant was not found in any database and was classified as deleterious by different missense prediction algorithms (Table 2). Cosegregation analysis showed that the patient's affected sister (III:3) also carried the variant (Fig. 3A). At the most recent assessment of the family (2016), patient III:5 had two children, IV:3, who was 4‐years old, and a son aged 15 months (IV:4). The same cosegregation analysis revealed that both children carried the pathogenic variant. Within this context, ophthalmic examinations were performed in order to comprehensively characterize the clinical features of these two family members. Interestingly, child IV:3 did show alterations in the most recent evaluation, revealing high hyperopia, an axial length of <21 mm in both eyes, nystagmus, and a small unilateral cataract in the right eye. The son (IV:4) did not show nystagmus but, similar to the case of IV:3, presented with hyperopia and a unilateral cataract in the right eye (Table 1). Fundus retinography was normal in both children.

Finally, the genetic assay of the family revealed that none of the unaffected parents carried the variant, at least in their blood DNA samples, suggesting the existence of germline or gonadosomatic mosaicism in one of them as the most likely scenario.

At the protein level, the identified mutation, p.(Arg44Pro), resulted in the substitution of a highly conserved amino acid of one of the two DNA‐binding domains of PAX6, the paired domain (PD) (Fig. 3D,E). In previous reports, the Arg44 residue was found to be mutated in two families affected by aniridia. In one case, a p.(Arg44Ter) nonsense change (c.130C>T) was identified in a Swiss family with several generations of affected individuals (Neuner‐Jehle et al. 1998); while in another, a p.(Arg44Gln) (c.131G>A) missense mutation was described in a patient of a Japanese pedigree (Azuma et al. 1998). In order to confirm the absence of the new p.(Arg44Pro) variant in the healthy population, we screened 180 control individuals. The analysis did not detect the presence of our variant in any control; however, we did identify p.(Arg44Gln) heterozygosity in three subjects. This, together with the fact that some vertebrates, such as the chimpanzee or the platypus, carry glutamine in this position, suggests that the previously identified variant p.(Arg44Gln) may be a polymorphism rather than a pathogenic mutation.

### *RBP4* gene mutation in complex microphthalmia

**Figure 4:**
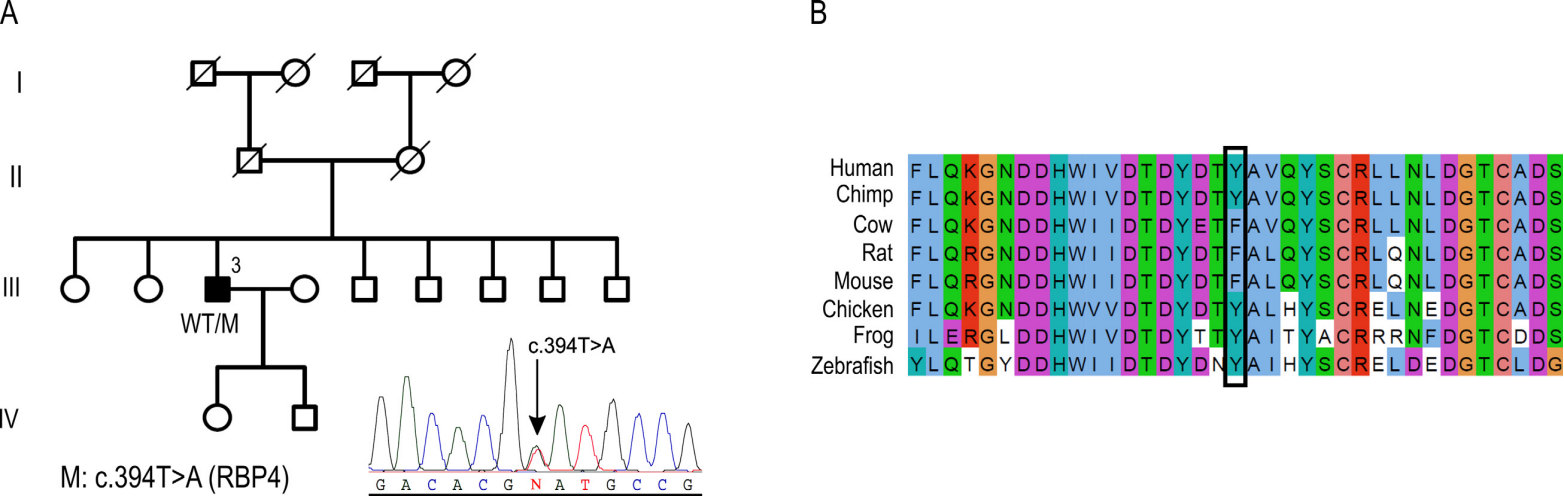
*RBP4* mutation associated with complex microphthalmia. (A) Pedigree of family MA_3 and chromatogram of the identified mutation. (B) Conservation of the Tyr132 residue at the protein level.

MA_3 is a Spanish family with a male individual displaying bilateral microphthalmia and coloboma affecting the iris and retina (Table 1 and Fig. 4A). There was no known previous family history of MA, none of the patient's seven siblings or offspring had the disease, and his maternal and paternal family came from the same region. Therefore, an autosomal recessive pattern was assumed as the most probable mode of inheritance in this case. For this reason, Sanger sequencing of coding regions of *STRA6* gene, which is responsible for autosomal recessive phenotypes compatible with the features of the patient, was performed. However, the analysis did not identify mutations in this gene. Subsequently, evaluation of the MA panel revealed that the patient (III:3) carried a heterozygous missense mutation in *RBP4*, c.394T>A (p.(Tyr132Asn)) (NG_009104.4, NM_006744.3, NP_006735.2). This variant was not described in any polymorphism or mutation database and was predicted as deleterious by all of the prediction programs used (Table 2). Screening of control individuals did not identify the mutation. Moreover, it affected a residue of one of the eight anti‐parallel beta strands of the RBP4 hydrophobic retinol ligand pocket, replacing a conserved hydrophobic amino acid (Tyr) with a polar uncharged hydrophilic one (Asn) (Fig. 4B). Cosegregation analysis was not possible in this case as none of the relatives agreed to participate.

## Discussion

In this work, we studied three unrelated families affected by different forms of MA. As previous single‐gene analysis had failed to identify the molecular cause of the condition, we were prompted to develop an alternative screening strategy based on the more recent NGS technologies. As such, we used WES to specifically analyze a panel of 47 MA genes. As these disorders show phenotypic heterogeneity, different modes of inheritance and clinical overlapping (Chassaing et al. 2014), we designed a panel that included all dominant, recessive, and X‐linked MA genes. Concerning the methodology, the use of targeted‐NGS was discarded, as it is limited in its flexibility for including new disease candidates, which are frequently being discovered in MA disorders. In this regard, WES strategy allows for rapid updating of the panel, as the inclusion of new genes is simply based on the modification of the filtering process during the bioinformatic analysis. In fact, two recently discovered MA genes, *PTCH1* and *OLFM2* (Chassaing et al. 2016; Holt et al. 2017), have already been added to a new version of the panel list.

Using the panel‐based WES analysis, a potential pathogenic mutation was identified in all three cases. Although a cohort consisting of only three cases is not statistically meaningful, the fact that relevant genetic mutations were found for all of the included families indicates that this particular strategy is a valuable diagnostic tool. Furthermore, it demonstrates that point mutation screening should be prioritized over gross deletion/duplication studies in nonsyndromic MA cases. Of the three families, two were initially thought to display autosomal recessive inheritance, due to their family history; however, the molecular assays subsequently revealed that they were in fact autosomal dominant cases showing complex inheritance patterns. Together, these facts highlight that a hypothesis‐driven approach based on the most probable pattern of inheritance in a particular family can lead to a skewed or even incorrect genetic diagnosis.

In our cohort, a mutation in the *OTX2* gene was identified as the molecular cause of bilateral anophthalmia (pedigree MA_1). This gene, however, has also been associated with other ocular phenotypes, including microphthalmia, optic nerve or optic chiasm hypoplasia, ocular coloboma and, more rarely, retinal dystrophies (Beby and Lamonerie 2013). Our results revealed that the affected patient of the family inherited the pathogenic variant from a healthy carrier parent. Although a de novo mutation in other genes not included in the panel cannot be ruled out as the molecular cause of MA_1 family, a case of incomplete penetrance seems to be the most plausible scenario. In fact, this form of penetrance has been previously described in several *OTX2* cases (Ragge et al. 2005a; Schilter et al. 2011; Gorbenko Del Blanco et al. 2012), as well as in other dominant and recessive MA genes (Williamson and Fitzpatrick 2014; Plaisancie et al. 2016a). This makes it difficult to estimate the risk of a child being affected by the condition and is an additional challenge for genetic counseling for MA. In addition to *OTX2*, high clinical variability was also observed in another MA candidate, *PAX6*, which has been previously associated with aniridia, microphthalmia, Peters anomaly, corneal dystrophy, congenital cataracts, and foveal hypoplasia (Azuma et al. 2003). In this study, *PAX6* was identified as the gene associated with microphthalmia, congenital cataracts, and foveal hypoplasia in family MA_2, which showed an extremely high intrafamiliar variability with regard to the clinical symptoms, affected ocular tissues, severity, and laterality. These findings are in accordance with previous reports in which variable phenotypes associated with *PAX6* were described within the same family (Vincent et al. 2004; Chograni et al. 2014). The third gene mutation identified in our cohort, in *RBP4*, was found in a patient with microphthalmia and coloboma (pedigree MA_3), as has been reported in only three other families (Chou et al. 2015). However, this gene has previously been identified as being associated with early‐onset retinal dystrophy (Cukras et al. 2012).

The reduced penetrance and variable phenotypic expressivity observed in MA genes may be explained by a number of different factors. Firstly, studies in *Otx2* ^+/−^ mice support the idea that the clinical variability is dependent on the genetic background (Hever et al. 2006), suggesting that modifier genes or particular polymorphisms (in *cis* or *trans*) may enhance or suppress the phenotype by controlling the gene transcription levels. Moreover, the complex gene expression networks in which transcription factors such as *PAX6* and *OTX2* participate, with several downstream targets, coactivators and corepressors regulated by both parental alleles, may result in phenotypic variation, even in cases with the same pathogenic variant (Yokoi et al. 2016). Secondly, epigenetic events or stochastic/environmental factors may also modify the final gene expression of these candidates, and thus the phenotypic outcome (Dipple and McCabe 2000; Ragge et al. 2005a). Finally, the observed variability between families could also be attributed to the type and localization of the mutation. In this regard, previous genetic analyses of *PAX6* have indicated that haploinsufficiency associated with heterozygous nonsense or frameshifting of the gene causes the classical aniridia phenotype, whereas most heterozygous missense mutations generate distinctive non‐aniridia phenotypes (Azuma et al. 2003; Vincent et al. 2003). This is in accordance with our findings, where we describe a novel missense *PAX6* mutation associated with microphthalmia and foveal hypoplasia. This affects the paired box domain of the protein, which may alter the ability of the mutant protein to bind some of the *PAX6* targets (Hanson 2003). This fact has been also observed in *RBP4*, in which recessive loss‐of‐function alleles have been associated with night blindness and retinal dystrophy, while heterozygous missense mutations have been described in dominant MA cases.

Besides the high degree genetic, allelic, phenotypic, and clinical heterogeneity, the occurrence of de novo mutations and mosaicism add even more complexity to the expressivity of MA genes, making the molecular diagnosis of such patients more difficult. In our cohort, a case of mosaicism was identified in the *PAX6* family (MA_2), in which the two affected siblings (III:3 and III:5) probably inherited the variant from a healthy mosaic parent. Gonosomal mosaic individuals transmitting the mutation to two affected offspring is not uncommon, as the number of precursor germ cells is thought to be small, thus increasing the risk of having a significant proportion of mutant cells (Leuer et al. 2001; Ragge et al. 2005a). Furthermore, a new mode of inheritance, based on skewed maternal transmission possibly modulated by dietary factors, has been recently described in MA. Chou et al. 2015 showed that in cases where a *RBP4* pathogenic variant is transmitted from the mother, the penetrance of the disease increased. Accordingly, the severity of MA and the genetic penetrance are comparatively low when transmitted from the father. This may be the case for family MA_3, as only one of the eight siblings had the disease, far from the expected 50% of affected offspring in an autosomal dominant case. Unfortunately, this could not be evaluated due to the unavailability of relatives’ samples; therefore, a de novo phenomenon or a case of regular incomplete penetrance cannot be ruled out.

In conclusion, here, we add three new variants to the mutation spectrum of MA, and describe complex inheritance patterns and phenotypic variability associated with these disorders. We also provide valuable clinical data that will contribute to the establishment of genotype–phenotype correlations, which is essential for gaining a better understanding of the molecular basis of these pathologies and their prognosis, and for guiding the management of MA patients.

## Conflict of Interest

The authors declare no conflict of interest.

## Supporting information


**Table S1**. List of genes included in the MA panel.
