# Supplementary material for: Panel‐based whole exome sequencing identifies novel mutations in microphthalmia and anophthalmia patients showing complex Mendelian inheritance patterns
Source: Mol Genet Genomic Med. 2017 Aug 21;5(6):709–19. doi: 10.1002/mgg3.329 (PMC5702572; doi:10.1002/mgg3.329)
Supplement: Supplementary file 1 — Table S1. List of genes included in the MA panel. [file MGG3-5-709-s001.pdf]

Supplementary Table S1. List of genes included in the MA panel

Gene	OMIM	Chromosome
ABCB6	605452	chr2
ALDH1A3	600463	chr15
ATOH7	609875	chr10
BCOR	300485	chrX
BMP4	112262	chr14
BMP7	112267	chr20
C12orf57	615140	chr12
CHD7	608892	chr8
COL4A1	120130	chr13
COX7B	300885	chrX
CRYBA4	123631	chr22
CRYGC	123680	chr2
FOXE3	601094	chr1
GDF3	606522	chr12
GDF6	601147	chr8
HCCS	300056	chrX
HESX1	601802	chr3
IKBKG	300248	chrX
MAB21L2	604357	chr4
MFRP	606227	chr11
NAA10	300013	chrX
NDUFB11	300403	chrX
NHS	300457	chrX
OTX2	600037	chr14
PAX6	607108	chr11
PORCN	300651	chrX
PRSS56	613858	chr2
PXDN	605158	chr2
RAB18	602207	chr10
RAB3GAP1	602536	chr2
RAB3GAP2	609275	chr1
RARB	180220	chr3
RAX	601881	chr18
RBP4	180250	chr10
SALL2	602219	chr14
SHH	600725	chr7
SIX6	606326	chr14
SMOC1	608488	chr14
SOX2	184429	chr3
STRA6	610745	chr15
TBC1D20	611663	chr20
TENM3/ODZ3	610083	chr4
TFAP2A	107580	chr6
TMEM98	615949	chr17
VAX1	604294	chr10
VSX2/CHX10	142993	chr14
YAP1	606608	chr11
